# Circulating Trimethylamine-N-Oxide and Risk of All-Cause and Cardiovascular Mortality in Patients With Chronic Kidney Disease: A Systematic Review and Meta-Analysis

**DOI:** 10.3389/fmed.2022.828343

**Published:** 2022-04-01

**Authors:** Zhongwei Zhou, Hao Jin, Huixiang Ju, Mingzhong Sun, Hongmei Chen, Li Li

**Affiliations:** ^1^Department of Clinical Laboratory, Yancheng Third People’s Hospital, The Sixth Affiliated Hospital of Nantong University, Yancheng, China; ^2^Department of Blood Transfusion, Yancheng Third People’s Hospital, The Sixth Affiliated Hospital of Nantong University, Yancheng, China; ^3^Department of Clinical Laboratory, Binhai County People’s Hospital, Yancheng, China

**Keywords:** trimethylamine-N-oxide, chronic kidney disease, dialysis, all-cause mortality, cardiovascular mortality, meta-analysis

## Abstract

**Background:**

Trimethylamine-N-oxide (TMAO) is expected to be a prognostic biomarker among patients suffering from chronic kidney disease (CKD). However, investigations on the association between TMAO and CKD prognosis are conflicting. In the present article, we aimed to assess the relationship of circulating TMAO with the risk of all-cause and cardiovascular mortality among CKD patients by a meta-analysis.

**Methods:**

Data were collected from PubMed, EMBASE, and Web of Science for systematically searching related literature (last update: February 2022). The multivariable-adjusted hazard risks (HR) and their 95% confidence intervals (CI) were pooled using random effects models.

**Results:**

Eleven prospective cohort studies covering 7,899 CKD patients were enrolled in this meta-analysis. When comparing individuals in the top and bottom baseline TMAO levels thirds, the multivariate adjusted pooled HR was 1.29 (95% CI 1.11–1.51, *P* = 0.001) for all-cause mortality, and 1.45 (95% CI 1.01–2.09, *P* = 0.043) for cardiovascular death. For continuous variables, per 1 unit increase of circulating TMAO levels was associated with a 3% higher all-cause mortality (HR 1.03, 95% CI 1.00–1.06, *P* = 0.032), but not significantly associated with cardiovascular death (HR 1.08, 95% CI 0.92–1.27, *P* = 0.346). Stratified analyses revealed that the positive relationship between TMAO and all-cause mortality remained significant after adjusting for diabetes, blood pressure, blood lipid, renal function, or inflammatory parameters.

**Conclusion:**

Higher circulating TMAO was associated with an increased mortality risk among patients with CKD, and this relationship may be dependent on TMAO dose and independent of renal function, inflammation, diabetes, hypertension, and dyslipidemia.

**Systematic Review Registration:**

[https://www.INPLASY.COM], identifier [INPLASY2021100049].

## Introduction

Chronic kidney disease (CKD) has become a global public health concern, influencing over 10% of adults worldwide (1). It is well documented that individuals with CKD confer a rising risk of cardiovascular incidents, cardiovascular mortality, and all-cause mortality (2). Effective kidney function tests and prognostic assessments are the important means for prolonging the survival of CKD patients. Currently, the estimated glomerular filtration rate (eGFR), albuminuria, and urine albumin-creatinine ratio (UACR) are commonly used for evaluating the prognosis of CKD in clinical practice (3, 4). However, the inadequacy of these markers are well documented. For example, older age, metabolic abnormalities and inappropriate sample preservation could affect the reliability of results (5, 6). Therefore, it is necessary to explore novel biomarkers for improving the prognosis of CKD.

In recent years, the interest in the correlation of the gut microbiome with health and disease is burgeoning, and that between intestinal flora and CKD is also a developing research area (7). Trimethylamine-N-oxide (TMAO), a small-molecule bioactive compound derived from intestinal microbial metabolism, has been attracting much attention recently (8). TMAO is normally present at low levels in circulating blood, and its abnormal or excessive accumulation can cause a wide range of diseases, such as diabetes, hypertension, cardiovascular disease, and CKD (9). Renal excretion is the primary route for TMAO clearance (10). When the kidney is diseased or injured, the accumulative TMAO can exacerbate renal inflammation and fibrosis, which further causes renal dysfunction (11). It is suggested that circulating TMAO levels were inversely correlated with eGFR, and TMAO concentrations decreased gradually with renal functional recovery in CKD patients after kidney transplantation (12, 13). A recent meta-analysis also revealed a negative association between TMAO levels and kidney function, which indicated that TMAO was positively associated with UACR, blood urea, creatinine, uric acid, and cystatin C (14). The abovementioned studies suggest that TMAO may be a potentially innovative prognostic biomarker for CKD individuals. To date, several studies have explored the associations between TMAO and the risk of all-cause and cardiovascular mortality among this population, but their findings are inconsistent. To address this issue, we performed this meta-analysis to sort out the literature on such associations.

## Methods

This review was performed following the Preferred Reporting Items for Systematic Reviews and Meta-Analyses (PRISMA) guidelines (15), with the protocol being registered at INPLASY (registration number INPLASY 2021100049).

### Search Strategy

Prospective observational studies were searched on PubMed, EMBASE, and Web of Science (last update: February 2022). Our search strategy was based on medical subject heading terms combined with text words: (trimethylamine N-oxide OR TMAO) AND (chronic kidney disease OR end-stage renal disease OR dialysis OR peritoneal dialysis OR hemodialysis) AND (mortality OR death). Furthermore, additional eligible studies were found by a manual searching of the references in related papers.

### Selection Criteria

The inclusion criteria for study selection are: (1) evaluating the relationship of circulating TMAO levels with all-cause and cardiovascular mortality; (2) targeting CKD patients regardless of dialysis; (3) reporting the multivariable-adjusted hazard ratio (HR) and the corresponding 95% confidence interval (CI); (4) targeting CKD patients having been followed up for over 1 year. We excluded studies (1) targeting patients with acute kidney injury or pre-kidney transplantation; (2) only reporting unadjusted risk estimates; (3) not written in English; (4) that were case reports, letters, conference abstracts, or unpublished studies.

### Data Extraction and Quality Assessment

Studies were screened by two researchers, with the information extracted including the information of the first author, publication year, study locations, patient type, gender distribution and age, sample size, comparison of circulating TMAO levels, events number, adjusted HR (95% CI), follow-up duration, and adjusted confounders. If a study reported multiple effect estimates based on several statistical adjusted models, the estimates in the most adjusted models were extracted. Any disagreements were resolved by a third author. The Newcastle-Ottawa Scale (NOS) was applied to the assessment of studies’ methodological quality (16). This scale has three domains of selection, comparability and outcome, offering maximum 9 points, from 0 to 3 denoting low quality, 4 to 6 denoting moderate quality to ≥7 denoting high quality.

### Statistical Analysis

Associations between TMAO and the risk of all-cause or cardiovascular mortality were reported differently in the included studies, such as per 10 μM increase, per log unit increase, per 2-fold increase, comparisons of high versus low, tertile, quartile, and quintile. To generate a consistent approach to this meta-analysis, the risk estimate was pooled by comparing the highest with the lowest third of the baseline TMAO levels. If some of the studies did not display effect measures as tertile, the risk estimate was converted according to the previously described methods (17–19). Briefly, the associations between the two was first assumed to be linear and the log-transformed HR was normally distributed. Then, the uniform scale was estimated as a scaling factor of 2.18 divided by 1.59 times the log HR for a comparison of high versus low, 2.18 divided by 2.54 times for the highest quartile versus lowest quartile, and 2.18 divided by 2.80 times for the top quintile versus the bottom. For studies (20–22) that reported per 1 log unit or per 10 unit change, they were first transformed into the standard deviation (SD) change, followed by 2.18 as a scaling factor. For one study (23) that reported per 2-fold increase, we assumed an approximate comparison between the top versus bottom tertile. With respect to two studies (24, 25) that reported both categorical variables and continues variables such as per 1 unit increase, continues variables were pooled separately. Meanwhile, in order to increase the pooled data, we also transformed the study (22) that reported per 10 unit increase to per 1 unit increase. A chi-squared test was conducted to assess studies’ statistical heterogeneity, with significance defined by <0.10. It was followed by the *I*^2^ test to quantify heterogeneity (26), with significance defined by greater than 50%. A fixed-effect model was used in the absence of significant heterogeneity, or a random effect model was adopted. For exploring heterogeneity sources and get more detailed data, a subgroup analysis was performed. The stability of the pooled data was assessed by a sensitivity analysis, where one study was eliminated sequentially at each turn. Egger’s test, together with a visual inspection of the funnel plot, was conducted for an evaluation of the publication bias. All of the statistical analyses were conducted on Stata 15.0 (StataCorp LP, College Station, TX, United States).

## Results

### Literature Search

**FIGURE 1:**
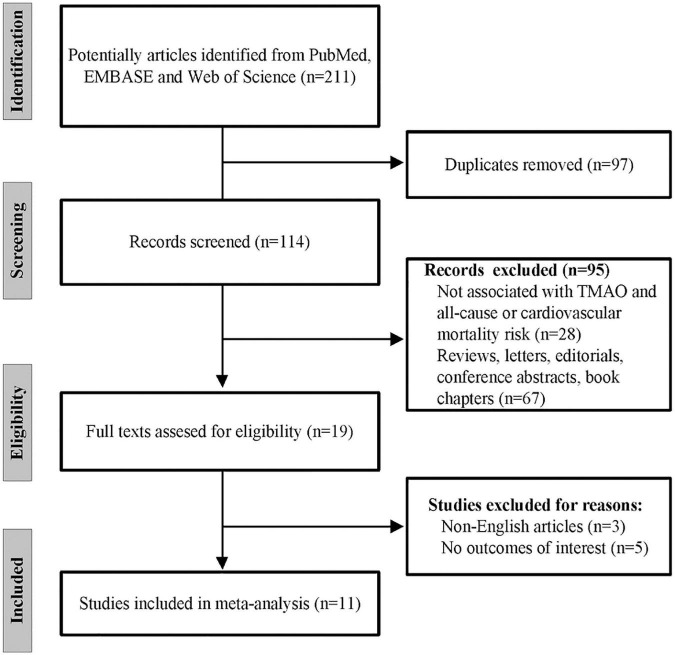
The flow chart of the study selection process.

A study selection flowchart is given in Figure 1, where 211 records in total were initially retrieved from PubMed, EMBASE, and Web of Science. Among them, 97 were removed due to duplication. Then, 95 records were further excluded after abstract and title screening. Of the remaining 19 records, eight articles were excluded owing to non-English languages and no outcomes of interest. Finally, 11 eligible studies (20–31) were included for later analysis.

### Characteristics of Selected Studies

Table 1 lists the baseline characteristics of the selected studies. Eleven studies, including six in United States, three in China, one in Sweden, and one in Netherlands, were published between 2015 and 2021 evaluated totally 7,899 CKD individuals. Four studies enrolled non-dialysis CKD patients, and four were limited to hemodialysis patients and two peritoneal dialysis patients, while one study included both non-dialysis CKD and hemodialysis patients. The number of included patients ranged 179–2133, and the proportion of male subjects ranged from 42.7 to 69.0%. Among these included studies, seven investigated serum TMAO levels and four investigated plasma TMAO levels. The age of CKD patients at baseline ranged from 48.0 to 70.0 years. The follow-up lasted 2.3–8.3 years. According to NOS quality assessment criteria, six studies were graded as high quality, and the rest were as moderate quality.

**TABLE 1:** Summary of clinical studies included in meta-analysis.

Author/year	Region	Types of patients	Sample size (% male)	Sample types	Age (years)	TMAO comparison	Event number/adjusted HR (95% CI)	Follow-up (years)	Adjusted confounders	Overall NOS
Kaysen et al. (27)	United States	HD	235 (55.3)	Serum	61.8 ± 14.2	Highest quartile 4 vs. lowest	Total death: 132; 1.14 (0.67–1.93)	4.0	Age, sex, BMI, race, diabetes, CRP, prealbumin, albumin	5
Tang et al. (28)	United States	Non-dialysis CKD	521 (48.0)	Plasma	70.0 ± 10.0	Highest quartile 4 vs. lowest	Total death: 174; 1.93 (1.13–3.29)	5.0	Age, sex, SBP, LDL-C, HDL-C, smoking, diabetes, log(hs-CRP), log(eGFR)	8
Robinson-Cohen et al. (29)	United States	Non-dialysis CKD	339 (69.0)	Plasma	57.3 ± 13.5	Highest tertile 3 vs. lowest	Total death: 45; 1.25 (0.48–3.28)	3.3	Age, sex, race, SBP, LDL-C, HDL-C, smoking, CRP, eGFR	7
Missailidis et al. (30)	Sweden	Non-dialysis CKD	179 (65.0)	Serum	55.0 ± 14.0	Middle + high tertile vs. lowest	Total death: 51; 4.32 (1.32–14.2)	5.0	Age, sex, diabetes, hs-CRP, GFR	4
Stubbs et al. (22)	United States	Non-dialysis CKD and HD	220 (42.7)	Serum	69.7 ± 10.3	Per 10 μM increase	Total death: NA; 1.26 (1.13–1.40)	4.0	Age, sex, BMI, race, eGFR, diabetes, hypertension, CKD stage, history of PCI, TC, TG, history of CABG, history of MI, history of CVA, history of PVD, history of CHF, smoking	6
Gruppen et al. (20)	Netherlands	Non-dialysis CKD	2133 (NA)	Plasma	NA	Per log unit increase	Total death: 219; 1.18 (1.02–1.36)	8.3	Age, sex, UAE	4
Shafi et al. (23)	United States	HD	1232 (43.3)	Serum	57.7 ± 13.8	Per 2-fold increase	Total death: 550; 1.06 (0.98–1.14); CV death: 216; 1.09 (0.96–1.24)	2.3	Age, sex, BMI, ICED score, cause of ESRD, SBP, albumin, relative volume removed on dialysis, residual kidney function	7
Stubbs et al. (31)	United States	HD	1243 (60.0)	Serum	54.0 ± 14.0	Highest quintile 5 vs. lowest	Total death: 458; 0.88 (0.64–1.20); CV death: 249; 1.05 (0.72–1.53)	5.3	Age, sex, BMI, race, SBP, albumin, dialysis vintage, BUN, history of smoking, MI, stroke, other CVD, PCI	8
Zhang et al. (24)	China	HD	252 (56.0)	Plasma	57.1 ± 14.5	High vs. low and per 1 unit increase	Total death: 123; 2.54 (1.71–3.76) and 1.14 (1.08–1.21); CV death: 39; 3.44 (1.67–7.08) and 1.18 (1.07–1.29)	6.1	Age, SBP, CHD, diabetes, cerebral infarction, cerebral hemorrhage, gout, calcium supplement, active vitamin D, albumin, prealbumin, hemoglobin, iron, hs-CRP	6
Fu et al. (21)	China	PD	1032 (57.0)	Serum	48.0 ± 14.0	Per log unit increase	Total death: 245; 1.22 (1.01–1.48); CV death: 129; 1.25 (0.95–1.62)	5.3	Age, sex, BMI, diabetes, history of CVD, MAP, albumin, TG, LDL-C, hs-CRP, rGFR, total Kt/V	7
Chang et al. (25)	China	PD	513 (58.1)	Serum	54.0 ± 15.5	Highest quartile 4 vs. lowest and per 1 unit increase	Total death: 142; 1.35 (0.79, 2.32) and 1.002 (0.999–1.004); CV death: 68; 1.94 (0.81, 4.67) and 1.002 (0.999–1.005)	5.3	Age, sex, BMI, diabetes, CVD, albumin, hs-CRP, potassium, phosphorus, residual kidney function, nPNA and calendar year of catheter implantation	7

*HR, hazard ratio; CI, confidence interval; SD, standard deviation; HD, hemodialysis; PD, peritoneal dialysis; CKD, chronic kidney disease; ESRD, end-stage renal disease; BMI, body mass index; TC, total cholesterol; TG, triglyceride; LDL-C, low density lipoprotein cholesterol; HDL-C, high-density lipoproteins cholesterol; BUN, Blood urea nitrogen; SBP, systolic blood pressure; CRP, C-reactive protein; hs-CRP, high-sensitivity C-reactive protein; GFR, glomerular filtration rate; eGFR, estimated glomerular filtration rate; rGFR, residual glomerular filtration rate; PCI, percutaneous coronary intervention; CABG, coronary artery bypass grafting; MI, myocardial infarction; CVA, cerebrovascular accident; PVD, peripheral vascular disease; CHF, congestive heart failure; UAE, urinary albumin excretion; ICED, Index of Coexistent Diseases; CVD, cardiovascular disease; MAP, mean arterial pressure; CHD, coronary heart disease; nPNA, protein equivalent of total nitrogen appearance normalized to body weight; NA, not available; NOS, Newcastle Ottawa Scale.*

### Associations of Trimethylamine-N-Oxide With the Risk of All-Cause Mortality

**FIGURE 2:**
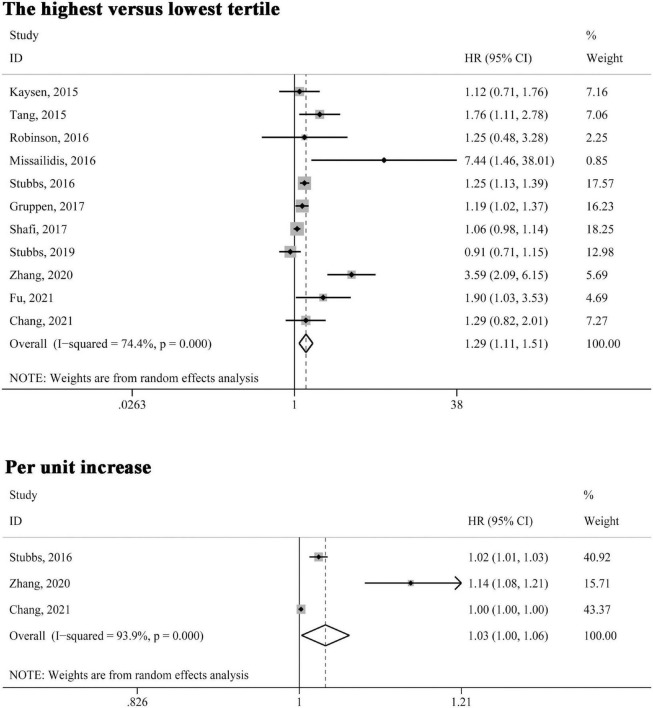
Forest plot of the highest versus lowest tertile and per unit increase in TMAO levels and all-cause mortality risk. HR, hazard risk; CI, confidence interval.

All included studies reported the association between circulating TMAO levels and all-cause mortality risk. As shown in Figure 2, when the association was presented as highest versus lowest tertile, the multivariable-adjusted HR was 1.29 (95% CI 1.11–1.51, *P* = 0.001); for three studies (22, 24, 25) in which the relationship was presented as per unit increase in TMAO levels, the pooled HR was 1.03 (95% CI 1.00–1.06, *P* = 0.032). Significant heterogeneity was found in both analyses (*I*^2^ > 50%, *P* < 0.10) and the random-effects model was selected.

### Associations of Trimethylamine-N-Oxide With the Risk of Cardiovascular Mortality

**FIGURE 3:**
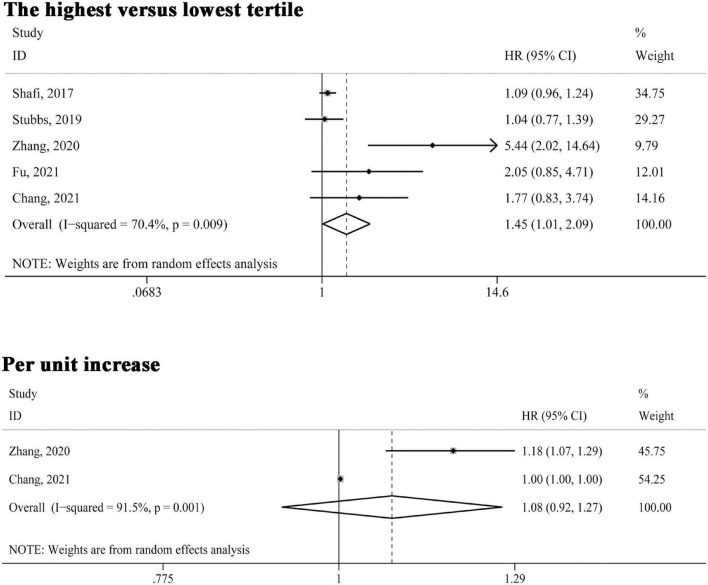
Forest plot of the highest versus lowest tertile and per unit increase in TMAO levels and cardiovascular mortality risk. HR, hazard risk; CI, confidence interval.

Five studies (21, 23–25, 31) reported the association between circulating TMAO levels and cardiovascular mortality risk. As indicated in Figure 3, in the comparison of highest versus lowest third of TMAO concentrations, the association was also significant (HR 1.45, 95% CI 1.01–2.09, *P* = 0.043); two studies (24, 25) reported cardiovascular mortality risk presented as per unit increase in TMAO levels, the pooled estimates were not statistically significant (HR 1.08, 95% CI 0.92–1.27, *P* = 0.346). Also, the random effect model was adopted given significant heterogeneity (*I*^2^ > 50%, *P* < 0.10).

### Subgroup Analysis

Due to small numbers of the included studies on cardiovascular mortality, only studies of all-cause mortality were subjected to a subgroup analysis. All the included studies were stratified according to geographic region, patient types, sample size, sample types, follow-up duration, whether statistical adjustment for race, BMI, blood pressure parameters or hypertension, hs-CRP or CRP, albumin and/or prealbumin, smoking, diabetes, kidney function, or blood lipid parameters. As shown in Table 2, stratified analyses indicated that there remained significant associations between TMAO and the risk of all-cause mortality in the subgroups adjusted for blood pressure parameters or hypertension (HR 1.30, 95% CI 1.05–1.60, *P* = 0.016), hs-CRP or CRP (HR 1.79, 95% CI 1.24–2.60, *P* = 0.002), albumin and/or prealbumin (HR 1.34, 95% CI 1.00–1.79, *P* = 0.46), diabetes (HR 1.68, 95% CI 1.23–2.31, *P* = 0.001), kidney function parameters (HR 1.19, 95% CI 1.05–1.36, *P* = 0.007), and blood lipid parameters (HR 1.36, 95% CI 1.13–1.65, *P* = 0.002). In addition, the associations seemed more significant for non-American patients, PD patients, and plasma samples. Nevertheless, although heterogeneity was reduced or disappeared in a few subgroups, it remained high in most subgroups.

**TABLE 2:** Subgroup analysis for all-cause mortality of the included studies.

Subgroup	No. studies	HR	95% CI	*P*	Heterogeneity	
					*I* ^2^	*P*
**Region**						
American	6	1.14	0.99, 1.30	0.069	61.5%	0.024
Non-American	5	1.92	1.17, 3.15	0.010	80.8%	<0.001
**Patient types**						
Hemodialysis patients	4	1.29	0.90, 1.84	0.163	85.8%	<0.001
Peritoneal dialysis patients	2	1.48	1.03, 2.12	0.036	0.0%	0.319
Non-dialysis patients	4	1.52	0.99, 2.34	0.057	58.1%	0.067
**Sample size**						
<500	5	1.76	1.07, 2.92	0.027	78.9%	0.001
≥500	6	1.16	1.00, 1.34	0.050	57.9%	0.036
**Sample types**						
Serum	7	1.16	0.99, 1.35	0.060	65.9%	0.007
Plasma	4	1.75	1.03, 2.97	0.038	82.0%	0.001
**Follow-up duration**						
<5 years	5	1.15	1.03, 1.29	0.013	40.8%	0.149
≥5 years	6	1.68	1.15, 2.48	0.008	83.5%	<0.001
**Adjustment for race**						
Yes	4	1.11	0.91, 1.36	0.292	47.4%	0.127
No	7	1.54	1.19, 2.00	0.001	81.6%	<0.001
**Adjustment for BMI**						
Yes	6	1.13	0.99, 1.29	0.062	59.7%	0.030
No	5	1.97	1.15, 3.40	0.014	80.9%	<0.001
**Adjustment for blood pressure parameters or hypertension**						
Yes	6	1.30	1.05, 1.60	0.016	83.7%	<0.001
No	5	1.33	1.02, 1.73	0.033	42.9%	0.136
**Adjustment for hs-CRP or CRP**						
Yes	7	1.79	1.24, 2.60	0.002	61.9%	0.015
No	4	1.12	1.00, 1.25	0.053	69.6%	0.020
**Adjustment for albumin and/or prealbumin**						
Yes	6	1.34	1.00, 1.79	0.046	80.0%	<0.001
No	5	1.29	1.10, 1.52	0.002	44.2%	0.128
**Adjustment for smoking**						
Yes	4	1.20	0.93, 1.54	0.157	63.9%	0.040
No	7	1.43	1.12, 1.83	0.004	79.3%	<0.001
**Adjustment for diabetes**						
Yes	7	1.68	1.23, 2.31	0.001	72.7%	0.001
No	4	1.08	0.98, 1.18	0.172	22.7%	0.275
**Adjustment for kidney function parameters**						
Yes	9	1.19	1.05, 1.36	0.007	63.2%	0.005
No	2	1.99	0.63, 6.22	0.239	90.5%	0.001
**Adjustment for blood lipid parameters**						
Yes	4	1.36	1.13, 1.65	0.002	17.3%	0.304
No	7	1.26	1.02, 1.57	0.033	79.0%	<0.001

*HR, hazard ratio; CI, confidence interval; BMI, body mass index; hs-CRP, high-sensitivity C-reactive protein; CRP, C-reactive protein.*

### Sensitivity Analysis and Publication Bias

**FIGURE 4:**
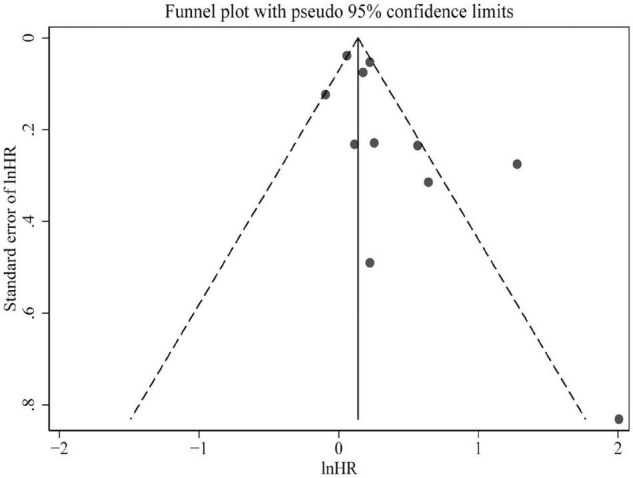
Funnel plot presented potential publication bias in the pooled estimate of all-cause mortality risk. HR, hazard risk.

**FIGURE 5:**
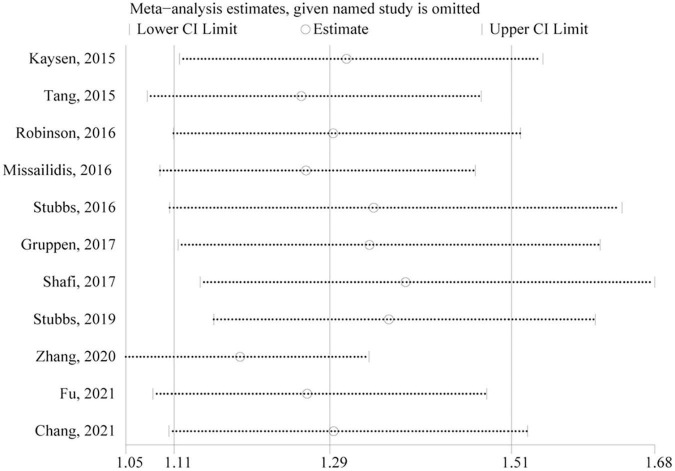
Sensitivity analysis of the association between circulating TMAO and all-cause mortality risk. CI, confidence interval.

Only studies of all-cause mortality were subjected to sensitivity analysis and publication bias. As indicated by sensitivity analysis, no single study significantly affected the overall pooled estimate (Figure 4). Egger’s test and a visual inspection of funnel plot were applied to the assessment of publication bias. The funnel plot was slightly asymmetric according to the visual inspection (Figure 5). Egger’s test further revealed mild publication bias may be present (*P* = 0.051).

## Discussion

To the best of our knowledge, this was the first systematic review and meta-analysis to explore the relationship of circulating TMAO levels with the risk of all-cause and cardiovascular mortality among CKD individuals. We found that CKD patients in the highest TMAO tertile had a significantly higher risk of all-cause or cardiovascular mortality compared with individuals in the lowest tertile. Limited data on continues variables suggested that per unit increase in TMAO was associated with a 3% increased risk of all-cause mortality, but not significantly associated with cardiovascular death. These findings suggest that higher TMAO is an independent predictor of mortality in CKD patients, and the relation is likely dependent on TMAO dose.

Recent studies have well established that individuals with impaired renal function had higher risks of cardiovascular events and all-cause mortality (2). Consequently, renal function-related parameters could be important confounders that influence the relationship between TMAO and mortality in this meta-analysis. Fortunately, however, most of the studies included in this meta-analysis had adjusted these indices, and we also observed a significant positive association between TMAO and all-cause mortality risk in the subgroup which included nine studies adjusted for kidney function parameters. This result suggests that the prognostic significance of circulating TMAO levels may be independent of some routine parameters of renal function among patients with CKD. In addition, CKD patients are usually accompanied by a series of comorbidities such as diabetes, hypertension, inflammation and lipid metabolism disorder (32). Most recently, several meta-analyses have been conducted to investigate the associations between TMAO and risks of adverse events such as hypertension, diabetes, and inflammatory factors, revealing that elevated TMAO levels were significantly positively correlated with these events (33–35). Similarly, to exclude the impact of these potential confounders on the association between TMAO and mortality, we also conducted subgroup analyses stratified by adjusting for these confounders and found that the relationship between TMAO and all-cause mortality risk remained significant in the subgroups adjusted for diabetes, blood pressure parameters or hypertension, hs-CRP or CRP, serum albumin or prealbumin, and blood lipid parameters. Since hs-CRP, CRP, serum albumin, and prealbumin are all inflammatory markers, these findings suggest that the prognostic value of circulating TMAO for predicting death in CKD may also be independent from inflammation, diabetes, hypertension, and dyslipidemia. Another meaningful finding in stratified analyses was that no association at a statistically significant level was found after adjustment for race or smoking, suggesting different races, and smoking habits may have implications for the link between TMAO and all-cause mortality. A selected study (23) showed that whites in higher TMAO levels had 4-fold and 2-fold higher risks of cardiovascular mortality and all-cause mortality, respectively, when compared with blacks.

Trimethylamine-N-oxide originates from its precursors called trimethylamine (TMA), a result of the fermentation by the gut microbiota of dietary nutrients, mainly including choline and carnitine (8). However, nutritional epidemiologic research failed to demonstrate that individuals consuming foods rich in choline and carnitine presented a health risk, such as cardiovascular events, or death (36). In contrast, an optimal dose carnitine supplementation was considered beneficial to improve exercise capacity and endurance in healthy individuals (37). Moreover, it has been suggested that moderate elevations in TMAO levels did not result in health problems, and the increased TMAO was considered risk factors for cardiovascular events or death only in individuals with insulin resistance and/or impaired renal function (38). One of the enrolled study (20) suggested that the association between TMAO and all-cause mortality was only observed in subjects with eGFR < 90 mL/min/1.73 m^2^, but not in the population with normal kidney function. Therefore, it may be advisable to explore the relationship between TMAO and diseases in a specific setting or population. However, the study populations included in the several meta-analyses were all mixed, consisting of both diseased and non-diseased cohorts (33, 34, 39, 40). In the present study, the individuals included in the pooled analysis were all CKD patients, and no healthy subjects.

This meta-analysis is subject to some potential limitations. First, potential heterogeneity sources were examined by subgroup analyses; however, heterogeneity was reduced or disappeared only in a few subgroups, but remained unexplained in most subgroups. Additionally, it is noteworthy that the results of subgroup analysis should be interpreted cautiously as each subgroup contained a decreased number of studies. Second, although only prospective studies with multivariate-adjusted risk estimates were included, the adjusted confounders varied with the included studies. For instance, one included study (20) adjusted only for age, sex and urinary albumin excretion. Therefore, inadequate adjustment for potential confounders in some studies may lead to biased risk estimates. Moreover, the converted effect estimate into extreme thirds in several studies might also lead to a certain degree of bias in our pooled analyses. Third, as only three studies were on all-cause mortality and two on cardiovascular death in pooling continuous variables (per unit increase in TMAO levels), these results may not be convincing enough to be applied in clinical practice. Four, there may be a mild publication bias in this meta-analysis. However, we should be fully aware that the significant heterogeneity across studies may also contribute to an asymmetric funnel plot (41). Moreover, our sensitivity analysis revealed that no individual article included in this meta-analysis significantly influenced the overall pooled results, suggesting the pooled effect estimates were relatively robust. Finally, dietary factors may affect circulating TMAO levels (42), while none of the included studies has adjusted risk estimates for diet-related variables. Hence, it is not clear to what degree the dietary factors influence risk estimates.

## Conclusion

To sum up, this meta-analysis demonstrated that circulating TMAO was independently correlated with the increased risk of all-cause or cardiovascular mortality in CKD patients, and the mortality risk may be dependent on TMAO dose. Another meaningful suggestion is that the relationship between circulating TMAO and mortality risk may be independent of renal function, inflammation, diabetes, hypertension, and dyslipidemia. More research efforts are still required to confirm the above findings.

## Data Availability Statement

The original contributions presented in the study are included in the article/supplementary material, further inquiries can be directed to the corresponding author.

## Author Contributions

ZZ and HaJ contributed to the writing of the manuscript. HuJ contributed to statistical analyses and quality assessment. MS and HC contributed to literature search and data extraction. LL contributed to the conception and design of the manuscript. All authors contributed to the article and approved the submitted version.

## Conflict of Interest

The authors declare that the research was conducted in the absence of any commercial or financial relationships that could be construed as a potential conflict of interest.

## Publisher’s Note

All claims expressed in this article are solely those of the authors and do not necessarily represent those of their affiliated organizations, or those of the publisher, the editors and the reviewers. Any product that may be evaluated in this article, or claim that may be made by its manufacturer, is not guaranteed or endorsed by the publisher.
